# HPV-KITE: sequence analysis software for rapid HPV genotype detection

**DOI:** 10.1093/bib/bbaf155

**Published:** 2025-04-10

**Authors:** Marek Nowicki, Magdalena Mroczek, Dhananjay Mukhedkar, Piotr Bała, Ville Nikolai Pimenoff, Laila Sara Arroyo Mühr

**Affiliations:** Interdisciplinary Centre for Mathematical and Computational Modelling, University of Warsaw, ul. Tyniecka 15/17, PL-02-630 Warsaw, Poland; Faculty of Mathematics and Computer Science, Nicolaus Copernicus University in Toruń, ul. Chopina 12/18, PL-87-100 Toruń, Poland; Interdisciplinary Centre for Mathematical and Computational Modelling, University of Warsaw, ul. Tyniecka 15/17, PL-02-630 Warsaw, Poland; Department of Biomedicine, University Hospital Basel, University of Basel, Hebelstrasse 20, CH-4031 Basel, Switzerland; Department of Clinical Science, Intervention and Technology, Forskningsgatan 56, Karolinska University Hospital, Karolinska Institutet, SE-14186 Stockholm, Sweden; Hopsworks AB, Åsögatan 119, SE-116 24 Stockholm, Sweden; Interdisciplinary Centre for Mathematical and Computational Modelling, University of Warsaw, ul. Tyniecka 15/17, PL-02-630 Warsaw, Poland; Department of Clinical Science, Intervention and Technology, Forskningsgatan 56, Karolinska University Hospital, Karolinska Institutet, SE-14186 Stockholm, Sweden; Research Unit of Population Health and Borealis Biobank, Faculty of Medicine, University of Oulu, Aapistie 5 B, FI-90014 University of Oulu, Finland; Department of Clinical Science, Intervention and Technology, Forskningsgatan 56, Karolinska University Hospital, Karolinska Institutet, SE-14186 Stockholm, Sweden

**Keywords:** DNA, RNA, HPV, NGS, sequence analysis, high-performance, Tversky, PCJ, Java

## Abstract

Human papillomaviruses (HPVs) are among the most diverse viral families that infect humans. Fortunately, only a small number of closely related HPV types affect human health, most notably by causing nearly all cervical cancers, as well as some oral and other anogenital cancers, particularly when infections with high-risk HPV types become persistent. Numerous viral polymerase chain reaction-based diagnostic methods as well as sequencing protocols have been developed for accurate, rapid, and efficient HPV genotyping. However, due to the large number of closely related HPV genotypes and the abundance of nonviral DNA in human derived biological samples, it can be challenging to correctly detect HPV genotypes using high throughput deep sequencing. Here, we introduce a novel HPV detection algorithm, HPV-KITE (HPV K-mer Index Tversky Estimator), which leverages k-mer data analysis and utilizes Tversky indexing for DNA and RNA sequence data. This method offers a rapid and sensitive alternative for detecting HPV from both metagenomic and transcriptomic datasets. We assessed HPV-KITE using three previously analyzed HPV infection-related datasets, comprising a total of 1430 sequenced human samples. For benchmarking, we compared our method’s performance with standard HPV sequencing analysis algorithms, including general sequence-based mapping, and k-mer-based classification methods. Parallelization demonstrated fast processing times achieved through shingling, and scalability analysis revealed optimal performance when employing multiple nodes. Our results showed that HPV-KITE is one of the fastest, most accurate, and easiest ways to detect HPV genotypes from virtually any next-generation sequencing data. Moreover, the method is also highly scalable and available to be optimized for any microorganism other than HPV.

## Introduction

Human papillomaviruses (HPVs) are one of the most diverse viral families infecting humans [1]. Fortunately, only a small number of closely related alpha HPV types are oncogenic. These infections, if persistent, are notorious for causing virtually all cervical cancers [2] and a fraction of oral and other anogenital cancers [3]. The need for accurate, fast, and efficient HPV genotyping has driven the development of numerous polymerase chain reaction (PCR)-based HPV DNA-targeted diagnostic methods [4]. However, detecting the correct HPV genotypes, particularly in cases of multiple infections, is often difficult due to the large number of genetically closely related HPV genotypes, whether using PCR or metagenomic sequencing.

Currently there are a wide variety of detection algorithms available for viral sequence detection, including sequencing alignment (BWA [5, 6], Bowtie [7, 8], MOSAIK [9]), protein alignment (Diamond [10], Kaiju [11]), marker gene detection (Metaphlan [12]), k-mer (Kraken2 [13], Clark [14]), and deep learning methods (Virtifier [15], VirFinder [16], DeepVirFinder [17], PPR-Meta [18], and ViraMiner [19]). Moreover, pipelines combining these tools have been established (HPVDetector [20], SearcHPV [21], V-pipe [22], ViromeScan [23], VirusFinder 2 [24], VirusSeq [25]). While detection methodologies are diverse, all these algorithms apart from direct reference mapping, are designed to perform multiple simultaneous reference genome comparisons with the sequence data. These methods are typically computationally intense and also prone to spurious detection if false positivity is not controlled. In addition, these methods often have specific memory and cpu requirements to be successfully employed.

There exist numerous pipelines for detecting HPV from sequencing data, as presented above. These pipelines do not use the possibility of parallel execution on multinode systems but only single-node threading. Moreover, they have multiple dependencies on third-party tools that have to be installed in the environment in the proper version, e.g. Bash shell, Perl, and Java.

In this method study we aimed to develop a novel viral sequence-based detection approach leveraging k-mer data analysis combined with the Tversky indexing [26]. Here, we assessed the HPV-KITE method, and its performance using three available datasets. This approach was developed and optimized for HPV detection from high-quality NGS sequence data. We demonstrate the methods efficacy through comprehensive benchmarking against standard HPV type and sequence-based detection methods. Our solution runs on a single node as well as on a multinode system with only a single dependency, which is Java 17.

## Material and methods

*HPV-KITE* (HPV K-mer Index Tversky Estimator application) is a quick and cost-effective method that enables rapid scanning of large genome datasets to identify HPV sequences. It was developed as a fast and accurate HPV detection algorithm for virtually any NGS sequence data, combining parallel computing with shingling and the Tversky indexing to detect any previously described HPV genotype.

The sequence datasets used for the development, calibration, and testing for HPV detection algorithm included a subset of the cancer genome atlas sequenced RNA-Seq dataset (TCGA, $N=894$, [27, 28]), another subset comprising sequenced RNA-Seq data ($N=300$) obtained from the Swedish cervical screening cohort [29] and another subset of DNA-based sequenced data ($N=82$) from the Finnish HPV vaccination cohort [30]. The application is written entirely using Java version 17 and is publicly available at GitHub (https://github.com/hpdcj/HPV-KITE, [31]).

### Sequence data indexing

**Figure 1:**
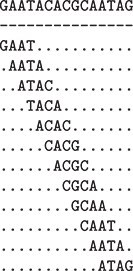
Shingling the GAATACACGCAATAG into four-character-long shingles.

The main concept of the HPV-KITE application is based on shingling. Shingling in this context refers to creating a set of shingles [32]—overlapping and nonoverlapping substrings of length k (k-mers, n-grams, subsequences) of the original sequence data. Figure 1 contains an example of four-character-long shingles produced from the sequence GAATACACGCAATAC. In the shingles set, we have 11 unique shingles: GAAT, AATA ($\times 2$), ATAC, TACA, ACAC, CACG, ACGC, CGCA, GCAA, CAAT, and ATAG.

Shingling was performed on the reference genomes (HPVs reference database in FASTA format) and the high-quality sequence data (FASTQ files) as follows.

First, the HPV reference genotype database was shingled. For this, one complete reference genome was retrieved for each known and validated HPV genotype (a total of 222 HPV types from the PaVE database [33], as of 23 February 2024) The curated reference genome dataset is provided as a FASTA alignment of all known HPV types. The database was shingled with the same shingle length as the intended input sequence data. Different shingle lengths were tested (between 9 and 110 nucleotides), with the default shingle length set to 16 for RNA-seq data and 31 for DNA sequence data.

Next, high-quality sequence data analyzed in this study were shingled. All reads within each paired-end FASTQ file were *concatenated* into a single, continuous string of DNA/RNA nucleotide sequence. Concatenated DNA reads were treated as an extended string of DNA nucleotide letters. These strings were then shingled to establish the unique DNA sequence patterns of the data.

With shingle sets from both the reference database and the input sequence data, the Tversky index was calculated as follows:


(1) \begin{align*}& S(X,Y) = \frac{| X \cap Y |}{| X \cap Y | + \alpha | X \setminus Y | + \beta | Y \setminus X |},\end{align*}

where X is a set of shingles from the input sequence (FASTA or FASTQ file), Y is a set of shingles from the HPV reference database or sequence data, $\alpha $ (alpha) represents the weight given to the importance of elements common to both sets X and Y, and $\beta $ (beta) represents the weight given to the importance of elements unique to set X or set Y. Parameters $\alpha $ and $\beta $ were set at 0 and 1, respectively, as the aim is to identify sequences in the FASTQ file (X) that match closely with known HPV sequences present in the reference database (Y), with only the elements unique to the reference database (Y) would contribute to the similarity score. Therefore, the equation can be simplified as


(2) \begin{align*}& C(X,Y) = \frac{| X \cap Y |}{| Y |}\end{align*}

Calculating the formula from the latter equation, the *coverage* (as number of unique shingles belonging to an HPV type present from the database) of the input sequence can be calculated. A threshold was set at $10\%$ viral coverage (covering $10\%$ of the HPV genome). Multiple HPV type infections were also considered. The exact number of covered k-mers depends on particular HPV virus and the shingle length. In the default, embedded HPV database, and the shingle length set to 31, the HPV213 has the lowest number of distinct shingles (7065), whereas HPV83 has the highest number of distinct shingles (8073), so the threshold $10\%$ means >706 and 807 shingles. On average HPV viruses have about 7484 shingles, and the median number of shingles is 7367.

### Datasets

#### Finnish cervical cohort

The only deep sequenced DNA metagenome dataset in this study comprised a set of FASTQ sequences ($>30$ mil. reads per sample) from 62 women, who originally provided cervical samples for the Finnish HPV vaccination trial [30]. These 62 samples were previously HPV genotyped using PCR-based Luminex HPV detection assay [34] and selected here for HPV-KITE testing purposes. The sequences were adapter and quality trimmed using *BBtools* and the preprocessed metagenomes were used both as raw (including human reads) and as human-filtered data (after filtering out human reads) to compare HPV-KITE results with the mapping based method of Diamond and standard PCR-based method for HPV genotyping (Luminex).

#### The cancer genome atlas

A subset of TCGA database, which comprises whole-genome sequences and the whole-genome transcriptomes of $\,{>}{20\,000}$ tumors, was used for algorithm scalability and sensitivity assessment [28]. A total of 894 sequencing bam files corresponding to RNA sequencing data from primary tumors from cervix uteri ($N=304$) and endometrium ($N=590$) were selected for the study and analyzed with HPV-KITE.

For accuracy assessment, HPV type detection was performed using Diamond (blastx) [35] for comparison. Briefly, original bam files included in the study ($N=894$) were already mapped to databases containing a human reference genome as well as other viral genomes. We therefore subjected all bam files to *pysam* v.0.16.1 (*htslib/bcftools* v.1.10.2 and *samtools* v.1.10) with a bed file containing human chromosomes and genomic regions to filter out the mapped human reads. Reads were quality checked and adapter trimmed with Trimmomatic [36] using 18 bp as minimal length for the reads. High-quality reads were screened against the human reference genome GRCh38 using NextGenMap [37] and only reads that did not map to the human genome, with identity over $75\%$ of their length, were considered as nonhuman, sorted, and converted to FASTQ format. Nonhuman reads were queried against all known established and non-established HPV protein sequences (from [33], accessed on 16 November 2023), using the Diamond blastx [35] with default parameters and -top 1. Samples were considered positive for HPV if a minimum of 10 reads were detected for any HPV type and if reads covered at least 750 bp of the viral genome with minimum identity of $90\%$.

#### Swedish cervical cancers dataset

RNA sequencing files belonging to a nationwide comprehensive study on HPV genotyping of invasive cervical cancers ($N=474$) [38] were also included in this study. HPV genotypes were identified after subjecting tumors to whole-genome sequencing and analyzed with Diamond (blastx) in the same manner as described for the TCGA dataset. Raw FASTQ files were subjected to HPV-KITE for calibration and accuracy assessment purposes.

### Experimental setup

All work and analysis were performed at CSC [39] using two supercomputers: *Puhti* and *Mahti*. Both supercomputers contain CPU nodes, with Puthi having a range of memory sizes as well as a large GPU partition and Mahti containing homogeneous CPU nodes, being meant for larger jobs (minimum 128 CPU-cores).

Puthi was used to process the Finnish cervical screening dataset, whereas Mahti was used to process the TCGA and the Swedish cervical cancer dataset. On Puhti, we used up to eight nodes. Each node had two Intel(R) Xeon(R) Gold 6230 CPU 2.10GHz (20 cores per socket, in total 40 CPUs) and 384 GB of RAM. On Mahti, we used up to 16 nodes, each with two AMD EPYC 7H12 64-Core Processors (64 cores per socket, for a total of 128 CPUs). For some benchmarking experiments, we further activated simultaneous multithreading (SMT), and increased up to 256 logical cores on each node. Each node was equipped with 256 GB of RAM.

#### Runtime configuration

The HPV-KITE application is available to download as a ZIP file from the official GitHub repository (https://github.com/hpdcj/HPV-KITE) as a release package [31]. The ZIP file contains two JAR files that have to be extracted into the current working directory. To run the application Java Runtime Environment (JRE), at least in version 17, is required.

The simple way to start the HPV-KITE application is just by typing:


\begin{align*} \mathtt{java}\ \text{-} \mathtt{jar\ hpv} \text{-} \mathtt{kite} \text{-} \mathtt{1.0.jar\ sample.fq.gz} \end{align*}

in the command prompt. It will start the processing of sample.fq.gz file using default parameters of HPV-KITE on the single node and output the result to the screen.

The application is also available as *conda package* [40] and can be installed by running the following command: conda install conda-forge::hpv-kite. Then the application can be executed by typing:


\begin{align*} \mathtt{hpv} \text{-} \mathtt{kite\ sample.fq.gz} \end{align*}

The more complex example with the SLURM Batch Job Script is presented in Appendix.

### Parallelization

Two ways of parallelization were considered: single and multiple nodes.

#### Single node

The initial approach considered parallelizing the reading of sequencing files across multiple threads and nodes. This approach is efficient for well-structured files, especially when using network drives. However, this approach needed to handle GZIP-packed .fq.gz files and varying line lengths within a single file, posing challenges. This issue was addressed by seeking a specific position in the file and searching for the first line containing the sequence to process. However, this would translate into a complex and error-prone implementation, as threads would need to calculate starting points to avoid skipping fragments or overlapping data. Moreover, jumping to the middle of a file requires at least reading it, incurring a performance cost.

Instead, a simpler solution for performance evaluation was tested. Here, a single thread read the input file, while shingling was delegated to threads in a thread pool. Data were submitted to the thread pool for processing when the input size exceeded a predetermined value (*processingBuffer*, default 16 MB). This approach proved highly efficient and was implemented with straightforward source code. It only required creating a ThreadPool object, defining processing tasks, submitting them to the pool, and monitoring task states using a Future<?> object. A FixedThreadPool was utilized with the default number of threads set to the available processors. Upon completing file reading, the master thread awaited the completion of all tasks. The master thread then conducted cross-checking of input shingles with each HPV virus from the reference database.

#### Multiple nodes

For parallelizing computations across multiple nodes, the PCJ library was chosen [41, 42]. This open-source BSD-licensed library, developed at ICM UW, implements the Partitioned Global Address Space (PGAS) programming model. It enables users to write parallel applications in Java that can leverage both multiple cores of a computer and multiple nodes of a cluster or even cloud. The PCJ library handles the initiation of execution across multiple nodes, utilizing mechanisms such as SSH connection or system-specific mechanisms like mpiexec, srun, or aprun. The PCJ library has also benn successfully used in bioinformatics for massively parallel sequence alignment with BLAST [43]. Moreover, applications developed with the PCJ library, including HPV-KITE, can run not only on supercomputers [44], cluster systems, or the cloud [45], but also on standard workstations and laptops, without requiring recompilation.

**Figure 2:**
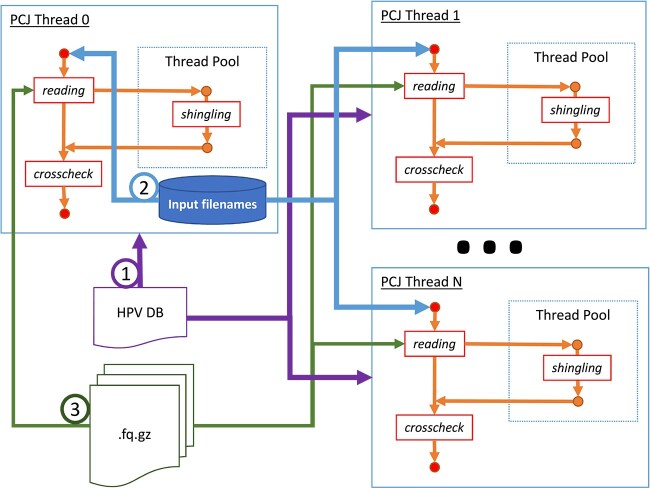
Schematic view of HPV-KITE execution.

The parallelization strategy employed in this algorithm involve dividing the workload between nodes, with each node hosting a single PCJ thread responsible for processing input files in parallel, utilizing local threads. Execution parameters, such as the HPV database file name, shingle length, *processingBuffer* value, and list of files, were communicated to the main process. The master thread (*PCJ Thread*-*#*0) organized a data structure to store the list of files to be processed, utilizing java.util.concurrent.ConcurrentLinkedQueue for its thread-safe properties. All threads simultaneously read the reference database. Initially, it was planned for the master thread to process the reference database and distribute it to other threads, but benchmarking revealed this approach to be slower. Instead, each PCJ thread requests the next file path from the master thread and processes files in sequence. Once a file is processed, the thread requests the next file path until all files are processed, terminating thereafter. Figure 2 presents the schema of the HPV-KITE execution.

### Performance assessment

The performance of HPV-KITE was evaluated by benchmarking its time and accuracy metrics against those golden standard HPV detection methods, and was performed in an OpenJDK Runtime Environment (version 21.0.2).

To assess and optimize time efficiency, the time needed to run the HPV-KITE was annotated for the Finnish cervical samples and the TCGA datasets using different number of nodes and threads and this time was compared with the time needed to run Diamond and Kraken2 within the same datasets (Diamond in version 2.1.6 on the Puhti system and Kraken2 in version 2.1.3 on the Mahti system). The database used for Kraken2 assessment comprised only virus reference sequences belonging to the HPVs (the same fasta files from PaVE that had been retrieved for building the databases for Diamond and for HPV-KITE).

For pipeline developing, optimizing purposes, scalability and accuracy assessment, HPV type results obtained from HPV-KITE were first compared with identified by Diamond using the Finnish cervical screening dataset, and with those provided by Diamond in version 2.1.9 for the TCGA dataset and the Swedish cervical screening cohort. This last cohort was also used for shingle length assessment.

We also run HPVDetector [20], VirusFinder 2 [24], and VirusSeq [25] pipelines using a subset of 36 nonhuman DNA metagenomes to benchmark HPV-KITE with the existing HPVs detection tools from metagenomes. We have omitted integration analysis part of each pipeline and we used only part for detection of HPV viruses.

## Results

### Parallelization evaluation

**Figure 3:**
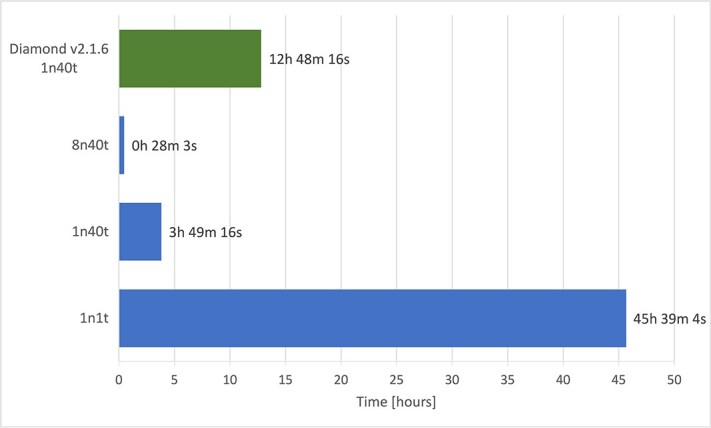
Total processing time for the whole Finnish dataset ($N=96$ samples) on the Puhti supercomputer using HPV-KITE and Diamond applications, where *XnYt* refers to *X* nodes and *Y* threads (threads in a pool) on each node.

The Finnish cervical metagenome dataset (FASTQ files, with or without human DNA) was subjected to Diamond and HPV-KITE using different number of nodes and threads (in a pool) on each node, to assess the need and amount of parallelization (Fig. 3). The findings indicated that relying solely on a single node and thread resulted in suboptimal performance, highlighting the necessity of parallelization.

Parallelization of generating shingles provided a big improvement in performance—the dataset was processed in about 4 h (11 times faster). Comparing that value with Diamond gave at least a 3x improvement in processing time. Moreover, the straightforward implementation of a multi-node solution decreased processing time; such a dataset was processed in less than half an hour. All these solutions used the 16 MB buffer for *GZIP buffer*, the 32 MB buffer for BufferedReader, and the 16 MB buffer for the processing lower limit (*processing buffer*).

### Scalability assessment

**Figure 4:**
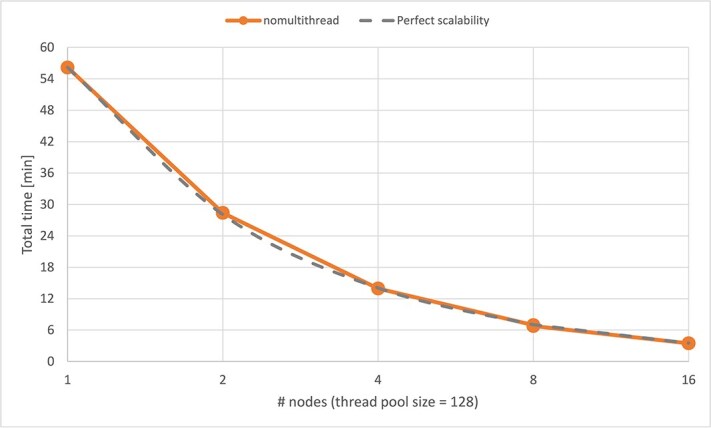
Processing time and scalability when processing the TCGA dataset with HPV-KITE using 1, 2, 4, 8, and 16 nodes on the Mahti supercomputer, each equipped with 128 threads in the thread pool.

Results on processing time indicated clearly that multiple nodes improved total time processing. We therefore aimed to assess the scalability of HPV-KITE when employing multiple nodes. The TCGA database consisting of 894 files (comprising 43 GB of nonhuman sequencing data) was subjected to the algorithm using 1, 2, 4, 8, and 16 nodes on the Mahti supercomputer, each equipped with 128 threads in the thread pool (Fig. 4). The relative scalability observed (in regards to processing time using one node) was ideal.

### Processing time compared with k-mer classification

**Figure 5:**
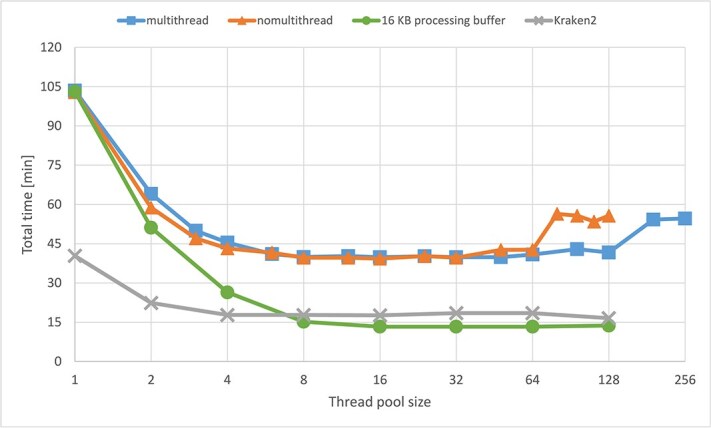
Time performance results for the TCGA dataset when subjected to HPV-KITE and Kraken2 on one Mahti node using default buffers sizes in regard to number of threads with activated and deactivated SMT; the 16 KB processing buffer is added to the graph (green color), as it revealed a four-times speed up when compared with the default buffer sizes).

**Figure 6:**
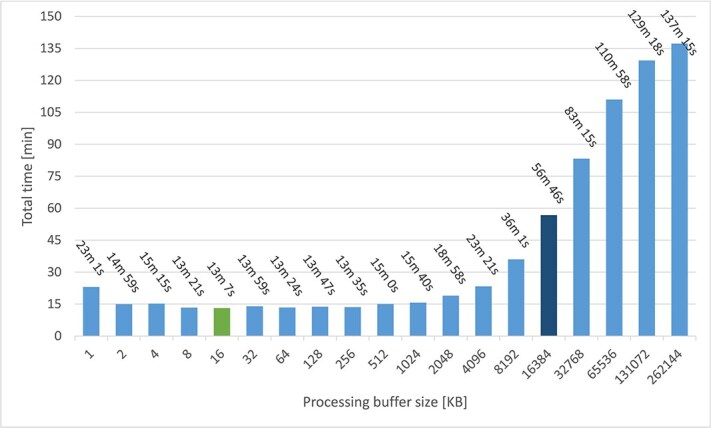
Processing time and buffer sizes for the whole TCGA dataset when subjected to HPV-KITE on one Mahti node and 128 threads in the thread pool; a dark blue bar presents previous default processing buffer size and a light green bar presents the current default processing buffer size.

As shingling (k-mer)-based classification is known to be faster than traditional mapping methods (Diamond), we subjected the TCGA dataset to both HPV-KITE and Kraken2 (broadly used algorithm based on k-mers) using only one node and compared activated and deactivated SMT used default buffers sizes (Fig. 5, orange and blue). We removed previously the human DNA, as it is a standard for Kraken2 analysis for processing time assessment. At first, HPV-KITE did not perform as well as Kraken2 and therefore buffer sizes were optimized (Fig. 6). Decreasing the buffer size 1024 times to 16 KB for the GZIP buffer, 32 KB for the reader buffer, and 16 KB for the processing lower limit (GZIP buffer equals processing buffer, and reader buffer doubles processing buffer size) gave a lower processing time than Kraken, showing four-times speedup, from >56 min to about 13 min (Fig. 6, green color and Fig. 5) when using a larger number of threads in the thread pool (visible from eight threads) (Fig. 6). However, it needs to be highlighted that enabling SMT does not speed up computational time (Fig. 5).

**Figure 7:**
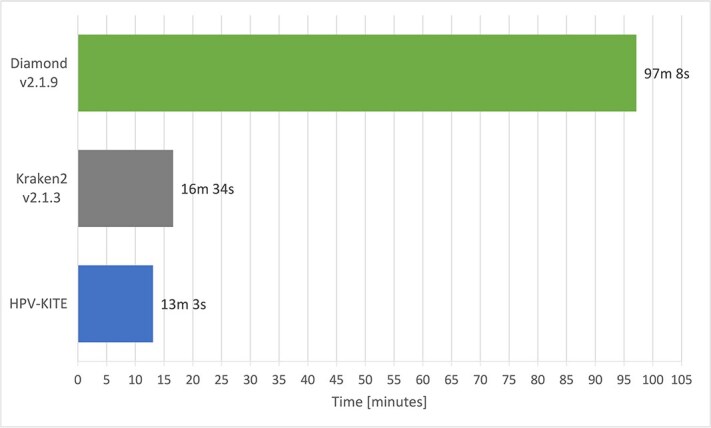
Total processing time for the whole TCGA study dataset using HPV-KITE, Kraken2, and Diamond applications; one Mahti node and 128 threads have been used for comparison.

The whole TCGA RNA dataset (including only nonhuman sequencing reads) was subjected to HPV-KITE, Diamond, and Kraken2, using the processing buffer size at 16 KB, one Mathi node, and 128 threads. The HPV-KITE successfully finished the process in 13 min, showing the best time performance, followed by Kraken2 (16 min, $25\%$ more time to process) and Diamond (97 min, 7x times longer) (cf. Fig. 7).

### Shingle length and threshold

For RNA dataset cutoff estimation (shingle size and threshold), the Swedish cervical cancer RNA-Seq dataset was used including all genomic material (human and nonhuman sequencing reads). Initially, a shingle length of 18 characters was selected arbitrarily, as it was the minimal length of the sequencing reads. To verify the correctness and accuracy of the proposed method, the results were compared with those obtained using a previously established method based on Diamond for detecting known infections [29]. The sample was considered accurately verified if the HPV type with the highest Tversky index value matched the previously known type, or if the sample was identified as not infected and the index value was below a specified threshold. Analysis of a subset of the TCGA RNA dataset (including human-derived reads, $N=200$) showed high accuracy ($98.7\%$) when compared with the mapping results (Diamond) and setting the threshold to 0.07 (translating into $7\%$ of the HPV).

**Figure 8:**
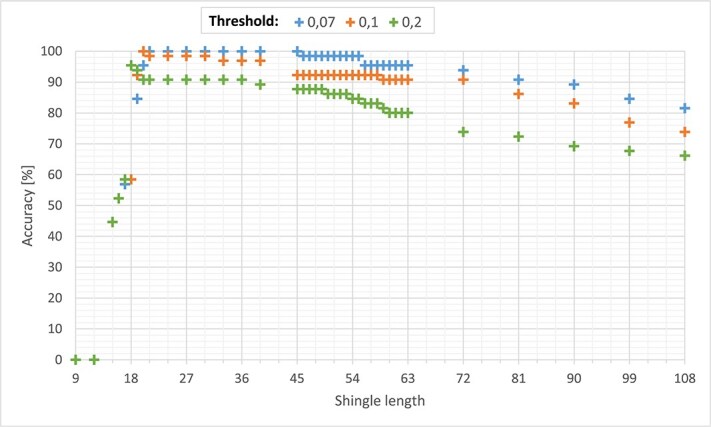
Shingle length, threshold, and accuracy assessment evaluated using the Swedish cervical cancer dataset.

To further optimize RNA sequence detection accuracy, both shingle length and thresholds were evaluated using the Swedish cervical cancer dataset. Best accuracy was obtained when settling a 0.07 threshold and a shingle length from 21–45 were or a 0.1 threshold and a shingle length of 20 (Fig. 8).

To assess HPV-KITE’s accuracy in DNA sequencing data (with longer sequencing read size than RNA datasets, and thus, expected higher accuracy to detect viral genomes), the Finnish cervical sample dataset ($N=62$) was used both with human DNA reads ($>30$ mil. reads per sample) and after removing human DNA reads from the metagenomes ($>{100\,000}$ reads per sample). HPV-KITE results were compared with those obtained with Diamond and PCR based HPV genotyping assay (Luminex). For HPV-KITE and using DNA metagenomes with PE 150bp read size a shingle length of 31 and a coverage threshold of $1\%$ were used. Our results revealed up to $96.2\%$ sensitivity for HPV-KITE to detect the same top hit HPV types and $100\%$ specificity to detect the HPV-negatives as was observed using direct mapping (Table 1). More comprehensively, when comparing the HPV-KITE results with the standard PCR-based HPV genotyping we obtained $88.9\%$ sensitivity to detect the same single and multiple (18 cases) HPV infections and $90.6\%$ specificity to detect the HPV-negatives when comparing with the PCR-based genotyping results (Table 2). Notably however, we excluded from the sensitivity analysis 12 metagenomes with <50 reads in total (out of $>30$ mil. reads per sample) mapped to any known alpha HPV. Among the samples with <50 reads mapped to any alpha HPV type, HPV-KITE could detect these genotypes but with $<1\%$ coverage.

**Table 1:** Sensitivity and specificity of HPV-KITE to detect the top hit HPV genotype compared with mapping results using Diamond; one case of single HPV55 infection detected by direct mapping but not observed as top hit by HPV-KITE; all likely HPV negative samples by direct mapping were also HPV negative by HPV-KITE.

		Diamond	
		positive	negative
HPV-KITE	positive	25	0
	negative	1	36
		0.962	1.000

**Table 2:** Sensitivity and specificity of HPV-KITE to detect all single and multiple HPVs infection positive cases (N=18, excluding cases with <50 reads mapped to any alpha HPVs) and compared with PCR-based genotyping using Luminex assay; one single HPV6 and one single HPV52 infection detected by PCR were not detected by HPV-KITE, while three PCR negative showed either HPV44 or HPV62 by HPV-KITE with at least $1\%$ detection coverage.

		PCR	
		positive	negative
HPV-KITE	positive	16	3
	negative	2	29
		0.889	0.906

Altogether, HPV-KITE results were the same regardless of using DNA metagenomes with or without human DNA; only results excluding human DNA are presented.

### Other pipelines

Several approaches already exist for HPV detection from metagenomic data and we tested HPVDetector [20], VirusFinder 2 [24], and VirusSeq [25] pipelines with the same metagenomes and compared these results with the ones obtained using HPV-KITE. The HPVDetector pipeline is a wrapper built around the BWA genome mapping [5, 6] and picard [46] tools. The full script is available from Dutt Lab [20]. We omitted the integration analysis part and just run the pipeline in *QuickDetect* mode for estimating the presence of particular HPV types DNA in the metagenomes available in our study. For the 36 nonhuman DNA metagenomes it took >2h (2h 27m 10s) for HPVDetector to run the samples while HPV-KITE needed <25min (22m 9s) to process the same metagenomes. VirusFinder was originally developed for detecting HPVs integrations into the host genome. Similarly to HPVDetector pipeline, we omitted the integration analysis part and ran VirusFinder 2 for above-mentioned samples. It took >7h (7h 3m 14s) for VirusFinder 2 to run the samples. VirusSeq pipeline is a wrapper built around the MOSAIK [9]—a reference-guided aligner for next-generation sequencing technologies. It was originally developed for detecting HPV genome integrations into the host genome rather than detecting any possible HPV DNA in a sample. Similarly to previous tools, we omitted the integration analysis part and run just the detection pipeline. It took >4 days (4d 3h 31m 12s) to process the same sample set as mentioned before.

Comparison of HPVDetector and VirusFinder with HPV-KITE revealed similar results; we were able to detect the same HPV type in a metagenome with VirusFinder as using the HPV-KITE and also detected with PCR-based Luminex methods. However, this only applied to the single HPV type infected cases, while only the most abundant single HPV type was detected using VirusFinder among the multiple HPV infected cases detected by HPV-KITE and PCR-based Luminex genotyping. This is likely due to the VirusFinder design to capture the single most abundant and integrated HPV types in a sample, and thus the method is limited when multiple HPV infections are present. All cases previously detected negative for HPV infections were also HPV negative with VirusFinder. In contrast to VirusFinder, HPVDetector detected most of the multiple HPV type infections, although often <50 reads per HPV type in cases with more than two HPV type infections. All cases previously detected negative using PCR-based Luminex genotyping were also HPV negative for these alpha HPVs with HPVDetector.

The last pipeline, VirusSeq, limits the detected viruses to only the most abundant HPV type. Moreover, the overall count of the mapped reads within a viral genome was set to 1000 as suggested by the authors of the tool as the empirical cutoff for virus existence. It results in only two positive detection and no detection of viruses for all other samples. However, in the working directory there exist intermediate results with multiple detected HPV viruses that can be used to check infection with lower cutoff value. Unfortunately, changing the cutoff value does not lead to the correct results in regards to PCR-based Luminex methods. VirusSeq has lower sensitivity—it does find only a fraction of detected HPV infections compared with HPV-KITE, even with lowered cutoff. Specificity is also low as there are few false positives compared with both PCR-based Luminex and HPV-KITE results.

## Discussion

Fast, simple, and accurate pathogenic detection methods for DNA and RNA sequence data are needed. Here, we present the HPV-KITE, a new method optimized for HPV DNA/RNA sequences detection based on shingling and the Tversky index for high-performance analysis of metagenome/metatranscriptome data.

HPV-KITE not only expedites viral detection but also maintains comparable sensitivity with existing approaches. Moreover, its ease of parallelization and reduced computational requirements make it an attractive prospect for streamlining the metagenomic pipeline analysis for HPV detection. Experimental results demonstrate that HPV-KITE achieves an accurate detection (sensitivity $>96\%$) of the most abundant HPV types in the DNA and RNA metagenome compared with the standard and computationally intense direct mapping algorithms, such as Diamond (Table 1).

More importantly, HPV-KITE achieved $89\%$ sensitivity and $91\%$ specificity to detect all single and multiple HPVs infection positive cases ($N=18$, excluding cases with <50 reads mapped to any alpha HPVs) and compared with PCR-based genotyping using Luminex assay (Table 2).

Additionally, HPV-KITE has significantly reduced the time needed to detect multiple HPV infections in the datasets compared with other presented solutions (Fig. 7 and Table 3).

**Table 3:** Running time for different pipelines and tools for a 36 nonhuman DNA metagenomes samples run on the Puhti supercomputer.

	Runtime
HPV-KITE	22m 9s
Kraken2	27m 54s
Diamond	1h 44m 17s
HPVDetector	2h 27m 10s
VirusFinder 2	7h 3m 14s
VirusSeq	4d 3h 31m 12s

One of the limitations of the HPV-KITE and any metagenomic approaches for HPV detection compared with the targeted PCR methods is the typically low amount of target genome reads available from a sample’s metagenome for the accurate target genome detection. A possible improvement of the method here is to incorporate the reverse complementary aspect of the DNA/RNA target into the shingles selection.

Previous studies have tried to estimate the threshold for reliable detection of HPVs from NGS data. Using HPV L1 gene targeted PCR followed with standard shotgun sequencing it has been proposed that at least $90\%$ identity and over $70\%$ coverage of L1 gene is needed for a positive detection of a particular HPV genotype [47]. For direct shotgun sequencing-based HPV detection at least 10 metagenome reads (reads size $\ge 70\text{bp}$) for a particular HPV type with minimum of $10\%$ genome coverage is suggested [48] but without confirmatory sequence similarity measures this threshold is already rather arbitrary with a risk of false-positive detection increasing. That is, a recent systematic study using PCR-targeted shotgun sequencing of HPVs estimated that positive HPV type call required a minimum of 127–212 DNA reads [49]. Similarly, when using nanopore sequencing the threshold was estimated as an average number of background reads plus 10 standard deviations [50]. Nonetheless, long-read sequencing technologies, such as Pacific Biosciences [51] and Oxford Nanopore Technologies [52], offer advantages in genome reconstruction, which could refine HPV assessment by improving genome coverage and reducing ambiguities in genotyping. Particularly, these technologies allow for more comprehensive assembly of HPV genomes, potentially reducing the need for stringent read thresholds in future analyses. Further studies are needed to determine how HPV-KITE could be adapted to long-read sequencing data, as the error profiles and read-depth requirements differ from short-read sequencing.

A notable technical limitation for HPV-KITE is that the program requires Java 17, while the default configuration on the Puhti supercomputer only includes Java 8, requiring a separate installation. Additionally, the user interface poses a challenge, as thorough instructions are needed for setup, and various technical issues may arise. To address these concerns, efforts are being made to improve accessibility through the HEAP platform [53, 54], a proposed solution designed to facilitate access to high-performance computing resources. This platform aims to allow researchers to run pipelines, including HPV-KITE, without the need for specialized infrastructure.

In summary, HPV-KITE is a rapid and accurate method for detecting well-characterized pathogen DNA/RNA, such as HPV types, from sufficiently deep metagenome or metatranscriptome sequence data. This method shows promise for detecting other microbial pathogens but requires further validation for bacteria and fungi detection. To detect other microbial pathogens other than HPV viruses, the one has to prepare database in FASTA file format and provide path to it using -DhpvVirusesPath=path parameter just before -jar parameter.

Moreover, to return more than three of the most abundant pathogens from the database, the -DoutputHpvCount=N parameter can be used—it can be limited to e.g. 10 pathogens or unlimited by providing 0 or any other nonpositive value. The output for each sample contains pathogens starting from the one with the highest Tversky index value and the next ones in descending order.

## Conclusion

The K-mer Index Tversky estimation is a method used in genetics to measure similarities between DNA sequences by analyzing length fixed short DNA/RNA sequences from the original data. HPV-KITE is a fast and accurate metagenome analysis method that significantly reduces computing time (from $20\%$ to even $700\%$) in comparison with standard mapping techniques such as Diamond and BLAST. It is highly scalable and can be easily used in parallelized environments. Moreover, the solution can be used even on a laptop without the need to use an advanced infrastructure on site.

Our results suggest that HPV-KITE has a potential for detecting not only HPV but also other organisms like bacteria and fungi. Further research is needed to assess its usefulness in metagenomics/metatranscriptomics analysis, which could broaden its applications in various research fields.

**Key Points**

- HPV-KITE is a novel method for rapid HPV genotype detection from DNA/RNA sequence data.
- HPV-KITE outperformed in time and scalability the standard sequence mapping and k-mer classification methods with similar detection accuracy.
- HPV-KITE enables fast and simple classification of target molecules for deep-sequence data virtually for any microorganism with a known genome information.

## Supplementary Material

hpv-kite_rev2_appendix_bbaf155

## Data Availability

Luminex PCR-based HPV genotypes from the Finnish cervical dataset was previously published in [34] and the corresponding nonhuman sequence data are available upon reasonable request from one of the author (V.N.P). Sequencing files belonging to the cancer genome atlas (TCGA) dataset are available at the public database (https://www.cancer.gov/tcga [28]). All sequencing analysis (HPV detection) belonging to Swedish Cancer cervical cancer (https://pubmed.ncbi.nlm.nih.gov/33020595/ [29]) dataset were published previously and the corresponding aligned, nonhuman sequences are publicly available at the Sequence Read Archive (SRA) within the bio-project ID PRJNA563802. All code for *HPV-KITE* is publicly available at https://github.com/hpdcj/HPV-KITE/ [31]. For efficient parallel data processing, the PCJ library was used. This open-source BSD-licensed library was developed at ICM UW and is available at https://github.com/hpdcj/PCJ/ [41].
