# Supplementary material for: HPV-KITE: sequence analysis software for rapid HPV genotype detection
Source: Brief Bioinform. 2025 Apr 10;26(2):bbaf155. doi: 10.1093/bib/bbaf155 (PMC11982018; doi:10.1093/bib/bbaf155)
Supplement: hpv-kite_rev2_appendix_bbaf155 [file hpv-kite_rev2_appendix_bbaf155.pdf]

1 Appendix

1.1 Starting HPV-KITE using SLURM Batch Job Script

To start calculation on the CSC Puhti supercomputer, the SLURM Batch Job Script presented in Listing 1 was used.

```
#!/bin/bash -l
#SBATCH --job-name=HPV-Kite
#SBATCH --account=project_NNNNNNN
#SBATCH --partition=small
#SBATCH --mem=8G
#SBATCH --nodes=4
#SBATCH --ntasks-per-node=1
#SBATCH --cpus-per-task=40

srun hostname -s \
       | sort > nodes.${SLURM_JOB_ID}

INPUT=$(ls /scratch/.../sequences/*.fq.gz)

srun -N ${SLURM_JOB_NUM_NODES} \
     -n ${SLURM_JOB_NUM_NODES} \
 /projappl/.../jdk-22.0.1/bin/java \
  -Xmx${SLURM_MEM_PER_NODE}M \
  -DnodesFile=nodes.${SLURM_JOB_ID} \
  -DoutputHpvCount=0 \
  -DfilesGroupPattern='P[0-9]+_[0-9]+_S[0-9]+' \
  -jar hpv-kite-1.0.jar \
   ${INPUT} \
  | tee output.${SLURM_JOB_ID}
```

Listing 1: SLURM Batch Job Script file

Lines starting with `#SBATCH` are job configuration, then the nodes file is generated with hostnames of all nodes associated with the job. As an input, all `.fq.gz` files from `/scratch/.../sequences/` directory is used. Then the application HPV-KITE is started on each node associated with the job. The results are written both into `output.*` file and associated with the job standard output stream using standard Linux tee command. Parameters used here and additional ones, as well as other ways to use HPV-KITE application are described in the project GitHub repository page [1].

References

[1] HPV-KITE GitHub homepage. https://github.com/hpdcj/HPV-KITE, Accessed: 2024-06-19.
